# Supported and valued? A survey of early career researchers’ experiences and perceptions of youth and adult involvement in mental health, self-harm and suicide research

**DOI:** 10.1186/s40900-019-0149-z

**Published:** 2019-04-29

**Authors:** Ruth Wadman, A. Jess Williams, Katherine Brown, Emma Nielsen

**Affiliations:** 1 Department of Health Sciences, The University of York, York, UK; 2 Institute for Mental Health, School of Psychology, University of Birmingham, Birmingham, UK; 3 Self-Harm Research Group, School of Psychology, The University of Nottingham, Nottingham, UK

**Keywords:** Involvement, Engagement, Knowledge exchange, PPI, Mental health, Self-harm, Suicide, Young people

## Abstract

**Background:**

Patient and public involvement (PPI) in mental health research, including self-harm and suicide research, is desirable (as with other health topics) but may involve specific challenges given the perceived sensitivity of the topic. This is particularly so when involving young people. We explore the experiences and perceptions of Early Career Researchers (ECRs) undertaking youth and adult involvement work in mental health, self-harm and/or suicide research. We consider current practice, barriers and facilitators.

**Methods:**

An online survey of a convenience sample of ECRs (*N* = 41) undertaking research on mental health, self-harm and/or suicide. Questions examined the perceived value of involvement work, involvement methods used, funding availability and the extent to which researchers felt knowledgeable, supported and confident in their involvement activities. Descriptive statistics are presented with appropriate tests. Open-ended questions, related to barriers and facilitators for involvement work, were subjected to an inductive thematic analysis.

**Results:**

Youth and adult involvement work were valued to a similar extent, though institutions were reported to value youth involvement to a lesser extent. Researchers’ knowledge, confidence and support ratings were comparable for youth and adult involvement. The involvement methods used with young people and adults were also similar, with analysing data being the least popular method used and developing resources (e.g. information sheets) being the most popular method used. Less than a third of participants reported that funding was available for their research involvement activities. Barriers to involvement in research on mental health, self-harm and suicide were: ethical issues and perceived risk; real costs (in terms of money/time) versus perceived value; and the challenge of recruiting people. Facilitators to involvement work were: expert examples, expertise and guidelines; and investment in involvement work.

**Conclusions:**

ECRs in the fields of mental health, self-harm and suicide are engaged in youth and adult involvement work. They value (find worthwhile) youth and adult involvement work to a similarly high extent, but feel their institutions may regard youth involvement slightly less highly than adult involvement. ECRs rate themselves as feeling similarly knowledgeable, confident and supported when doing involvement activities with both age groups. Nonetheless, significant barriers to involvement work on these topics are reported and are generally issues that need to be tackled at an institutional level (ethical/governance issues and lack of funding).

## Plain ENGLISH summary

Talking to people about mental health can be hard, but it is important that researchers involve people with mental health difficulties (including self-harm and suicidal behaviour) in their research. It may be particularly challenging to involve young people in research on these topics, but involvement activities can help make research more appropriate and user-friendly. We asked researchers at an early stage in their careers (‘Early Career Researchers’) about their experiences of involving young people and adults in their research on mental health, self-harm and/or suicide. Forty-one researchers completed an online survey.

The Early Career Researchers valued youth and adult involvement work to a similar extent, but they said that their universities valued youth involvement in research less than adult involvement. The researchers felt similarly knowledgeable, confident and supported in doing research involvement activities with young people and adults. Young people and adults were involved in research in similar ways. They were most often asked to develop research materials (such as participant information sheets), and least often involved in analysing research data. Fewer than one in three researchers had funding available for their involvement activities. The researchers said that barriers to involving people in research were: 1) the worry that people might be harmed or upset by such activities; 2) the financial cost and time spent on involvement activities; and 3) the challenge of finding people to be involved in the research. We suggest that Early Career Researchers need more help from universities to support their research involvement work, including better links with people in the community and more time and money.

‘Public involvement’ in research, defined by the National Institute for Health Research INVOLVE guidelines as *“research being carried out ‘with’ or ‘by’ members of the public”* [1] is increasingly a requirement for UK health research. Public involvement in research includes “*… working with research funders to prioritise research, offering advice as members of a project steering group, commenting on and developing research materials and undertaking interviews with research participants”* ([1], p 6). In contrast public ‘engagement’ is used to refer to providing and disseminating information and knowledge about/from research (though this can overlap and sometimes be used interchangeably with ‘involvement’), and ‘participation’ refers to people taking part in a research study. [1]. For clarity, we use the term ‘involvement’ throughout, but distinguish youth involvement from adult involvement. This allowed us to identify any specific challenges experienced when trying to involve young people in research.

The importance of involvement in mental health research is recognised by researchers, research participants and stakeholders in the NHS and voluntary sector [2, 3]. The general public are stakeholders who hold valuable knowledge and expertise, and this includes young people. Well-planned public involvement work can: (i) focus research aims and methods, making research more relevant, (ii) enhance research participation (e.g. in marginalised groups), and (iii) lead to research impact ([1, 4]. In the field of mental health, public involvement in research can address stigma and raise awareness. Arguably, people have a democratic right to be involved in research which affects them (and which is often taxpayer funded). However, this work is frequently inadequately supported - being underfunded and undervalued [5]. Unfortunately, this work can also be seen as tokenistic by members of the public involved [6], and particularly young people [7].

Early Career Researchers (ECRs) can be broadly defined as researchers who have yet to secure a permanent academic post in a Higher Education Institution, and can include postgraduate students (Masters and Doctoral level), postgraduate and postdoctoral researchers (Assistants, Associates and Fellows) and newly appointed lecturers. ECRs are arguably at the vanguard of public involvement work in research, though little is known about their experiences or perceptions of undertaking such work. There is a need to understand current practice, successes and challenges in order to inform and support the research involvement work undertaken by this group.

Youth and adult involvement work is increasingly utilised in mental health research [8]. There is evidence from a longitudinal examination of the Mental Health Research Network study portfolio database (NIHR Clinical Research Network) that those studies that involved patients to a greater degree were more likely to have achieved their recruitment targets, and thus be regarded as successful [8]. There is also evidence of personal benefits to those who take part in involvement activities in mental health research, such as: (i) gaining knowledge, understanding, expertise and the development or refreshing of skills (e.g., communication skills, interpersonal skills), (ii) increased self-confidence and a sense of belonging and being valued, and (iii) and a reduction in personal fears and self-stigma (where an individual agrees with/internalizes negative public attitudes towards mental illness) [6, 9, 10]. Public involvement can enhance researcher knowledge and improve research delivery [5, 11]. Involvement work informing mental health research priorities has highlighted some interesting discrepancies between the priorities of services users compared to those of professionals (e.g. calls for more psychosocial research rather than biomedical research) [12]. However, while the benefits of utilising service user perspectives when developing mental health services are often recognised, there is a tendency to overlook service users when planning research and setting its priorities [10].

Research evidence relating to youth involvement in mental health research is particularly scant. A qualitative study of young people involved in mental health research suggests that young people want to actively contribute *throughout* the research process and that meaningful research involvement was perceived as making a difference in ensuring research projects were relevant [13]. This study highlighted specific training needs for researchers in order to achieve this. Recently, it has been demonstrated that youth involvement in research can generate original, credible and useful findings that reflect both young people’s perspectives and inform the extant literature. Examples include participatory research approaches in developing self-care strategies for mental health [14] and participant-researchers co-producing a Transition Preparation Programme for Child and Adolescent Mental Health Services (CAMHS) [15]. A commentary paper describing the experience of being a young participant-researcher within CAMHS highlighted that young people felt valued and gain confidence from meaningful, creative and well-supported involvement activities [16]. However, the authors also cautioned that researchers may underestimate the amount of time needed to effectively support young people to maximize their roles as co-researchers, citing, for example, recruitment and governance problems [15, 16]. Anecdotally, we have heard that involving young people in research on mental health, especially topics viewed as sensitive - such as suicide and self-harm - can be perceived as particularly ‘risky’, and thus challenging, to undertake. Through this study, we aimed to explore these challenges in more detail. Similar challenges have been documented when involving young people in alcohol and drug research [17]: professionals and services can act as gatekeepers and thus serve as barriers to involvement; it can be challenging to keep young people engaged with involvement activities and requires a flexible and sensitive approach.

### The present study

Mental health research, including research into self-harm and suicide, is likely to present specific challenges to youth and adult involvement work (i.e. working with ‘vulnerable’ groups on topics deemed to be ‘sensitive’ in nature). The present study examines the perceptions of ECRs in these fields regarding youth and adult involvement. Specifically, we examine the experiences and perceptions of ECRs who have been involved in youth and/or adult involvement work in the field of mental health, including self-harm and suicide research, in order to: (i) determine the extent to which these ECRs feel that work in youth and adult involvement is valued, (ii) describe how ECRs in mental health involve young people and adults in research, and (iii) identify common barriers and facilitators in undertaking youth and adult involvement in their research. We hope to provide valuable and practical information about current practice, barriers and facilitators, with the aim of finding ways to better support youth and adult involvement work in mental health research.

## Method

### Participants

The survey was open to ECRs undertaking research in mental health, self-harm and/or suicide in the UK (a convenience sample). A total of 41 participants completed the survey. Due to the small research field and familiarity of those working in this area, no additional identifiable information was collected (e.g. age, gender, current institution). Status as an ECR was self-determined and thus included postdoctoral and postgraduate researchers (amongst others).

### Design and procedure

The anonymous self-report questionnaires were administered online. Participants were recruited via e-mail listings, a link on a website for ECRs (Early Career Researchers Youth And Public engagement on Self-harm Network) and via social media. As such, the total number of potential participants reached is unknown. The survey was made available in February and March 2017. Ethical approval was given by the departmental Research Ethics Committee (January 2017).

### Youth involvement in this study

‘Youth Speak’ were a mental health research advisory group operating between 2013 and 2017 at the lead author’s institution (at the time of data collection). The members were aged between 14 and 24 years old and had contributed to a number of mental health research projects over the 4 years. The group was established upon the principle of developing a youth culture that promoted inclusiveness and collaboration, and as such the members were not asked about, or expected to disclose, their mental health difficulties.

The Youth Speak group were involved in the planning and content of a symposium for ECRs on youth and adult involvement in mental health research. The plans for the symposium were discussed at a Youth Speak meeting. Due to timing and financial constraints, the group members were not able to attend the symposium. Instead, we produced a short film presentation of the young people talking about their experiences of research involvement and engagement activities that was shown at the symposium. Ideally, it would have been advantageous for the Youth Speak group members to attend the symposium, particularly as they considered teaching researchers (including ECRs) how to work effectively with young people to be an important part of their Youth Speak role. Informal feedback regarding the symposium suggested that the ECRs would also have welcomed the opportunity to meet and discuss research involvement with the Youth Speak group members.

### Measures

The survey included closed- and open-ended questions about perceptions and experiences of youth and adult involvement in research. The survey questions were piloted at the symposium for ECRs engaged in research on mental health, self-harm and/or suicide (December 2016). Following piloting, a small number of changes were made to the survey questions, including the addition of a question about knowledge of youth and/or adult involvement activities undertaken by the respondents’ employing institution and an open-ended question about what would facilitate youth and adult involvement work in research. The final survey included four sections. Note that questions were asked regarding youth and adult involvement separately, though no specific definition of youth versus adult (i.e. age range) was given, reflecting the broader lack of consensus regarding the definition of ‘youth’. The survey questions and response options are given in Table 1.

**Table 1:** Survey questions and response options

	Question	Response options
Research and job details	Position/job title	Undergraduate student, Postgraduate taught Masters student, Postgraduate research Masters student, PhD student, Postgraduate researcher (research assistant/associate post without PhD), Postdoctoral researcher (research associate/fellow with PhD), Lecturer, Other (please state)
	Number of years of research experience in total	
	What is your research primarily concerned with? ^a^	Mental health, Self-harm, Suicide, Other (please state)
	What is your youth/adult involvement work primarily concerned with? ^a^	Mental health, Self-harm Suicide, Other (please state)
	Does your supervisor/line manager undertake youth and/or adult involvement work?	Yes, No, Don’t know
	Does your institution(s) undertake youth and/or adult involvement work?	Yes, No, Don’t know
Value of youth/adult involvement	To what extent do you personally feel that youth/adult involvement is valuable?	1 (not at all valuable) to 10 (extremely valuable)
	To what extent do you personally feel YOUR current youth/adult involvement work is valuable?	1 (not at all valuable) to 10 (extremely valuable)
	To what extent do you feel the organisation(s)/institution(s) you are associated with value youth/adult involvement?	1 (not at all valuable) to 10 (extremely valuable)
	To what extent do you feel the department(s) you are associated with value youth/adult involvement?	1 (not at all valuable) to 10 (extremely valuable)
Current youth and adult involvement in research	Please indicate all of the ways in which young people and/or adults are involved in your current work ^a^	Identifying research topics, Prioritising research questions, Preparing research applications, Design of research, Management of research (e.g. steering/advisory group), Developing participant information resources, Undertaking/analysing research (e.g. member of research team), Contributing to the reporting of the study report, Dissemination of research findings, Other (please state)
	How often are young people and/or adults are involved in YOUR work?	Never, Rarely, Sometimes, Often, Always
	How often are young people and/or adults are involved in YOUR ORGANISATION’s work?	Never, Rarely, Sometimes, Often, Always, Don’t know
Barriers and facilitators	What are the biggest barriers to increased youth and adult involvement in your research?	Free response
	What would help you to better undertake youth and adult involvement work in your research?	Free response
	Is funding available for your youth and/or adult involvement work?	Yes No
	If yes, what is the source of this funding?	
	How confident do you feel when engaging with young people and/or adults during work related to mental health/self-harm/suicide?	1 (not at all) to 10 (extremely)
	How supported do you feel when engaging with young people and/or adults during work related to mental health/self-harm/suicide?	1 (not at all) to 10 (extremely)
	How knowledgeable do you feel when engaging with young people and/or adults during work related to mental health/self-harm/suicide?	1 (not at all) to 10 (extremely)

Note. For most questions, responses were given for youth involvement and adult involvement separately. The terms ‘value’, ‘confident’, ‘supported’ and ‘knowledgeable’ were not defined, therefore it was for individuals to interpret the terms themselves

^a^Respondents could select more than one response option

We did not provide respondents with definitions of any of the terms used in the questions. For instance we did not define what was meant by the word ‘value’ in the questions asking the extent to which the respondents/their organisation/their department value youth/adult involvement. We anticipate that our respondents understood the use of the term ‘value’ as meaning ‘held in high regard’/‘seen of worthwhile pursuit’, although it is possible that other interpretations (e.g. regarding monetary worth, or principles and beliefs held) were applied.

### Data analysis

Quantitative descriptive statistics (analysed using SPSS V24 for Windows) are presented. Responses to open-ended questions concerning barriers and facilitators were analysed thematically using inductive coding. The coding mapped onto the questions asked, so that distinct ‘barriers’ themes and ‘facilitators’ themes were generated. The initial analysis was done by AJW and KB, with codes being reviewed and developed further by EN and RW, before the final coding was refined collectively by the group. To establish the reliability of the initial coding, 17 unlabelled extracts were presented alongside the theme descriptions to an independent coder, PR, who was blind to the original analysis and coded on their own. PR’s coding was then compared to the original coding, with good reliability (the independent coder attributed 82.4% of the extracts to the same codes as the original coders). Following this, a final framework was synthesised from the previous themes, which was reviewed by all authors to ensure clarity.

## Results

Participant details regarding research interests and experience are given in Table 2. The reported range of research experience was 1 to 13 years (*M* = 5.41; *SD* = 3.34). Thirty-four participants were able to state their current contract length. The majority of respondents (*n* = 13) held three-year contracts. This is likely a reflection of the high proportion of PhD students comprising our sample (*n* = 20; 48.8%). Only four respondents had permanent contracts. The primary research focus of participants was mental health, followed by suicide, self-harm, and ‘other’ (*n* = 15; 36.6%), which included research topics such as memory, prisons, and intervention development. Many ECRs had overlapping areas of research interest, indicated by selecting two or more options (*n* = 18; 43.9%).

**Table 2:** Participant details

	Percentage (frequency)
Position/job title	
Undergraduate student	0.0% (0)
Postgraduate taught/research Masters student	9.8% (4)
PhD student	48.8% (20)
Postgraduate researcher	9.8% (4)
Postdoctoral researcher	19.5% (8)
Lecturer	9.8% (4)
Other	2.4% (1)
Research focus	
Mental health	65.9% (27)
Self-harm	29.3% (12)
Suicide	41.5% (17)
Other	36.6% (15)
Focus of youth/adult engagement and involvement work	
Mental health	61.0% (25)
Self-harm	24.4% (10)
Suicide	39.0% (16)
Other	34.0% (14)
Supervisor/line manager undertake youth and/or adult involvement work?	
Yes	70.7% (29)
No	12.2% (5)
Don’t know	17.1% (7)
Institution(s) undertake youth and/or adult involvement work?	
Yes	82.9% (34)
No	2.4% (1)
Don’t know	14.6% (6)

### Youth and adult involvement in Research

Participants’ research interests were reflected in their research involvement work: this was primarily concerned with mental health, then suicide, self-harm and ‘other’ (see Table 2). The majority of participants had a supervisor or line manager who was also engaged in youth or adult involvement research activities. Similarly, most participants reported that their institution undertook such work.

**Fig. 1:**
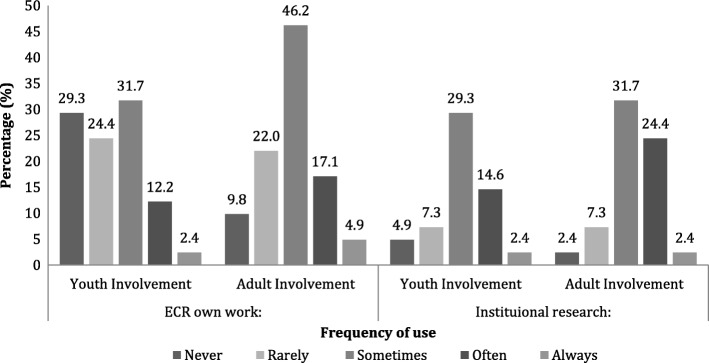
Frequency of youth and adult involvement work reported for ECR and their institution *Note.* For the responses regarding frequency of involvement work within institutions, 41.5% of responses for youth involvement were ‘don’t know’ and 31.8% of responses for adult involvement were ‘don’t know’

The reported frequency (‘never’, ‘rarely’, ‘sometimes’, ‘often’, ‘always’) with which young people and adults were involved in the respondents’ research and their institutions’ research is compared in Fig. 1. Adult involvement was reported more frequently than youth involvement within the ECR’s own work, whereas these values were more similar for institutions.

Table 3 summarises the value, support, confidence and knowledge ratings given by participants with respect youth and adult involvement work. Median ratings of the value of youth versus adult involvement work were the same for ‘ ECR values involvement’ and ‘department values involvement’. ECR confidence median ratings were also the same. A Wilcoxon Matched Pairs test showed that the participants rated youth and adult involvement to be similarly valuable (*Z* = − 1.14, *p* = 0.26) despite the differences in the frequency of the groups being involved in work (see Fig. 1). For the majority of the remaining ratings, youth involvement work was rated less highly than adult involvement work, although these differences were non-significant (‘ECR values *their* involvement’ (*Z* = − 1.48, *p* = 0.14); ‘ECR feels supported’ (*Z* = − 2.14, *p* = .03), and ‘ECR feels knowledgeable’ (*Z* = −.92, *p* = .36)). It is worth noting that the range of responses here for youth involvement covered the full range of the scale, suggesting experiences were more varied. When ECRs were asked about their institutions’ value of involvement, there was a significant difference between youth involvement (Mdn = 7.00) and adult involvement (Mdn = 8.00), (*Z* = − 2.88, *p* = .00).

**Table 3:** Perceived value, support, confidence and knowledge ratings relating to youth and adult involvement work

	Youth involvement				Adult involvement			
	Min	Max	Median	IQR	Min	Max	Median	IQR
ECR values involvement	5.0	10.0	10.0	8.0–10.0	4.0	10.0	10.0	9.5–10.0
ECR feels their involvement is valued	3.0	10.0	7.5	6.0–10.0	3.0	10.0	8.0	6.0–10.0
Institution values involvement	1.0	10.0	7.0	6.0–10.0	3.0	10.0	8.0	6.0–9.0
Department values involvement	2.0	10.0	7.0	5.5–10.0	3.0	10.0	7.0	6.0–9.5
ECR feels supported	1.0	9.0	6.0	4.0–7.0	1.0	10.0	7.0	4.0–8.0
ECR feels confident	1.0	10.0	7.0	5.3–8.0	3.0	10.0	7.0	6.0–8.0
ECR feels knowledgeable	1.0	10.0	6.0	5.0–8.0	3.0	10.0	7.0	5.0–8.0

*Note.* Higher scores indicate higher ratings of feeling valued, supported, confident and knowledgeable with regards to youth and adult involvement

Participants reported the ways in which adults and young people were involved in their research projects (Table 4). Similar patterns were seen across types of involvement methods for both populations. However, higher frequencies were generally seen for adult involvement, as compared to youth involvement. This was particularly notable in methods such as identifying research topics and disseminating research. There were no significant differences between the frequencies of methods that were reportedly used for youth and adult involvement work (McNemar’s tests for each method).

**Table 4:** Methods of research involvement

	Youth (%)	Adult (%)
Identifying research topics	24.4	43.9
Prioritising research questions	24.4	34.1
Preparing applications	12.2	19.5
Study design	24.4	41.5
Management (e.g. advisory group)	24.4	39.0
Developing resources	31.7	46.3
Analysis	4.9	9.8
Contributing to reporting of study	14.6	19.5
Dissemination	26.8	46.3

Analysing data was the least utilised method of both youth and adult involvement. Across both populations, developing resources was the most popular method. Additional methods reported (in an ‘other’ response option) were involving people in developing or giving feedback on research tools, and research involvement groups training other people in research involvement skills.

### Funding

Less than a third of participants (*n* = 13, 31.7%) reported that funding was available for their research involvement activities. Sources included research councils/funding bodies (e.g. as part of doctoral grants) or internal university funds. Thus, despite limited funding, ECRs managed to conduct research involvement (with only some utilising a variety of funding sources).

### Barriers and facilitators

A total of five main themes describing challenges (three themes) and facilitators (two themes) to youth and adult involvement work were identified in the open-ended question responses. Two of these themes contained subthemes. The themes and subthemes identified are described below with example quotes.

#### Ethics and risk

##### Ethical approvals and access to young people/specific groups

A key barrier to involvement work centred on getting ethical permission for work to be conducted, which could impact on the researchers’ ability to access the groups for involvement activities, *“Ethics. Access to young people, as a student.” (ID:36)*. Specific issues identified with ethical governance procedures included safeguarding concerns and length of time to secure approvals, *“Issues regarding consent/safeguarding” (ID:16); “Long ethics processes to contact people through the NHS” (ID:24).* Negotiating ethical issues within institutions was described as “*… costing a lot of time and effort to the researcher” (ID:4)*. Some respondents simply responded to the question of barriers to research involvement with a one-word answer *“ethics” (ID:02, ID:06)*, so it was unclear whether this referred to the process of gaining ethical approval for their involvement work, or referred to the broader ethical considerations at play. However, as noted by one respondent, excessive concerns about the riskiness of involvement activities in self-harm or suicide research appears to influence the ethics procedure they need to negotiate: *“Ethics committees are risk averse to the detriment of research and rather than weight up the implications of a study case by case, they try to manage the risk with blanket policies” (ID:04)*.

##### Perceptions of risk involved

Involvement work with young people and adults was described as ‘risky’ by several respondents, with groups with lived experience of self-harm and/or suicide being viewed as ‘vulnerable’ and involvement work potentially affecting well-being ( “*… concern that talking about suicide will make them suicidal” [ID:25]*, *“Organisations aren’t willing to engage for fear of perpetuating suicide” [ID:08]*). This was especially salient for work involving young people, *“Particularly for youth, I think they are grossly mis-underestimated. They are often thought to be too vulnerable to be involved.” (ID:39)*. These perceptions were reported to influence both individuals involved in the research (e.g. supervisors) and organisations: “*My PhD supervising committee think that people who have attempted suicide should not be interviewed*. *They think I should interview stakeholders instead” (ID:01)*; *“Departments which have never had researchers with a focus on self-harm or suicide are disproportionately worried about perception of the research and of reputation issues” (ID:04).*

## Real costs (in terms of money/time) versus perceived value

Costs in terms of *“time” (ID:18, ID:21)* and *“funding” (ID:40)*, incurred by involving young people and adults in the life-cycle of research, were identified. Though keen to undertake involvement activities, a lack of money for such work was a big issue for the ECRs: *“Money money money - if I had more funding I would include PPI in all my PhD studies. Unfortunately, I don’t” (ID:20)*. These costs, alongside a perceived lack of benefit from involvement work, were highlighted as a barrier to youth and adult involvement in research. *“A lack of financial resources, support, and awareness about how valuable this involvement is to the target population and organisations.” (ID:22).* The respondents indicated that research involvement is *“Not taken seriously” (ID:10)* and “*… is often not held in the same esteem as other research activities” (ID:5)*. There was thus a tension between ECRs undertaking (potentially costly) involvement work, when such work was not seen to be valuable at an institutional level: *“There is scant institutional support for putting this [PPI] into place and without this and without the funding to do it, it’s very difficult to implement by ourselves.” (ID:37)*.

### The challenge of recruitment

Several responses indicated that difficulties in recruiting young people and adults were a barrier to involvement work. Some responses reflected difficulties in recruitment generally in the field *“Recruitment of participants” (ID:34), “Finding the relevant people” (ID:16), “No links with societies, charities etc, therefore difficult recruitment …*” *(ID:7), “Reaching a representative enough group” (ID:29).* Others highlighted challenges regarding working with specific populations “*… I work with a population who by definition are ill, and so their own illness trajectory can get in the way for [these] individuals” (ID:9)*.

### Expert examples, expertise and guidelines

Several respondents indicated that they would find it easier to conduct involvement work if they had the opportunity to learn from others with more experience: *“Collaborations with experienced colleagues” (ID:7); “Access to learn from more experienced researchers about how they raise awareness of the opportunity for involvement, and the different ways in which the [y] involved people in their work outside of the traditional, steering group type set-up, which for me doesn’t quite cut it.” (ID:17)*. The merits of professional resources that could be used, such as training schemes and research involvement guidelines, were also highlighted *“Having a framework or model to base engagement on.” (ID:15), “Possibly more training on engaging people on a sensitive topic such as suicide” (ID:8).*

#### Investment in involvement work needed

##### Practical resources, especially money

Whilst costs were identified as one barrier to youth and adult involvement in research (see above), increasing resources was suggested to promote the practice of involvement work. Increasing the availability of financial resources was commonly mentioned: *“Funding specifically for this [involvement work].” (ID:16); “Grants!” (ID:3); “Small grants to help fund workshops” (ID:29); “Availability of funding at an early stage” (ID:40).* One respondent felt that research councils should do more to financially support involvement work, *“I think every funding council providing PhD funding should give each student a PPI pot of money” (ID:20)*. Other practical resources required to facilitate involvement activities included time (*“More dedicated time, to listen and incorporate young people’s views.*” *[ID:12]*) and technology ( “*… e.g. virtual meetings by teleconference/videoconferencing and the facilities to do this” [ID:9])*.

##### Wider community support

A factor noted by respondents as potentially facilitating youth and adult involvement in research was if involvement efforts were supported more by those in the wider community, *“If families and educational institutions support this involvement.” (ID:25)*. The role that researchers may play in changing wider communities’ behaviour and/or beliefs was noted, *“increasing gatekeepers’ understanding of the importance of these topics (i.e. the extent of the problem of suicide and self-harm)” (ID:41)*. Improvement in the relationships between community groups and researchers was mentioned as a facilitative factor *“Improved links with the local community” (ID:4), “having a dedicated PPI lead with good links into local youth networks” (ID:30)*. One respondent suggested *“A network that could facilitate access to young people and the public via existing groups without the need to specifically recruit them for your trial/study” (ID:14)* would be useful, with another noting the need for *“access to groups which are not NHS based” (ID:24)* for mental health involvement work.

##### A supportive culture within institutions

Some participants indicated that their ability to conduct meaningful involvement work would be benefited if they received greater support for involvement projects from the organisations they work within, in ways unrelated to practical resources. A shift in culture was highlighted as being important at an individual level (i.e. more support from staff), the departmental level, and at a broader institutional level: *“My supervising committee understanding where I am coming from in my research, rather than putting up barriers.” (ID:1); “My current department really does not support public engagement at all. Trying to change this!” (ID:39); “Openness to youth involvement in actual research process.” (ID:35); “Research culture which values youth and public involvement” (ID:11).*

## Discussion

This paper reports findings from a survey of ECRs regarding their perceptions and experiences of involving young people and adults in research on mental health, self-harm and/or suicide. The findings suggest that although involvement work is valued, researchers at an early stage in their career face particular - and potentially significant - barriers in undertaking this work. Some differences in involving young people compared to adults were apparent - for example, young people were reported to be involved in the ECRs’ own research less frequently.

### Perceived value of youth and adult involvement in research

ECRs considered youth and adult involvement in research to be valuable generally, as well as being valuable to their own research. They also reported that their institution and department appeared to value youth and adult involvement in research. However, a notable proportion of ECRs reported that their institution did not undertake involvement activities (or were not aware of such work). Ratings suggest that ECRs feel relatively well supported, confident and knowledgeable when undertaking youth and adult involvement in research. Although some ratings were lower for youth versus adult involvement, the only rating difference of significance was the perception of value that institutions gave to youth versus adult involvement (lower for youth). Youth involvement yields the same barriers as adult involvement, but may have additional barriers such as gatekeeping, ethical concerns about child involvement, and being considered a ‘difficult to reach’ population in general. It is also possible that young people are regarded as being less capable of making a valuable contribution to research, but this requires further exploration. It should be noted that most respondents had a supervisor or line manager who undertook youth and/or adult involvement, thus providing a potential source of expertise and support.

### How ECRs involve young people and adults in research on mental health, self-harm and suicide

Both young people and adults were involved in research through similar methods across the research life cycle. Both groups were most frequently reported to be involved in developing research resources (such as participant information sheets) and dissemination of research findings. These are relatively discrete and time-limited activities within the lifespan of a research project (and arguably easier to manage and cheaper). The least often reported involvement activities for young people and adults were data analysis, preparing research applications and reporting the research (a more in-depth level of involvement along the co-production continuum) - tasks which possibly require more expert knowledge of research topics and academic processes. Youth and adult involvement in the analysis or reporting of research findings (for example, respondent checking and validation of themes in qualitative work) may not always be achievable or desirable, depending on the methodology. Indeed, this specific method might have a limited capacity within youth involvement (e.g. due to complexity of tasks and lack of research experience), however, arguably it is the responsibility of the researcher to make such activities accessible to the target audience. Public involvement in preparing research applications should arguably be more common practice given funding body PPI requirements, but it should be acknowledged that ECRs may not have been involved with their current research at such an early stage. Finally, compared to adults, young people were reported to be less frequently involved in identifying research questions, contributing to study design and in managing the research (in the form of an advisory committee, for example) - although our data do not speak to why this might be. If young people are not being involved in setting research questions and priorities, their views risk being ignored in the national youth mental health research agenda [12]. It might be that researchers are less inclined to engage with youth involvement due to the additional steps needed to adjust tasks to meet the abilities, preferences or language needs of younger people.

A previous analysis of the types of involvement activities reported in applications to the National Research Ethics Service (NRES) also found that dissemination of research findings was the most frequently reported activity whereas analysis was the least frequently reported activity [3]. Young people involved in mental health research have expressed a desire to be involved in every stage of the research life-cycle [13] and at the far-end of the involvement/participation continuum children can be successfully supported as researchers in their own right through child-led research [18, 19]. Future research could explore ways of facilitating youth involvement in mental health research further, for example, identifying those parts of the research process in which young people can be involved and where youth involvement can lead to improvements [20].

### Barriers and facilitators in undertaking youth and adult involvement in mental health, self-harm and suicide research

Barriers to involving young people and adults in research on mental health, suicide and self-harm were: 1) ethical issues and perceived risk (ethical approvals and access to young people/specific groups, perceptions of risk involved); 2) real costs (in terms of money/time) versus perceived value; and 3) the challenge of recruiting people. The barriers related to ethics and perceived risk may help explain why youth involvement was reported less frequently or why young people were less often involved in particular involvement activities (e.g. managing research). It is concerning to see that ethical considerations appear to be presenting a significant barrier to involvement in research, particularly in light of recent guidance from the Health Research Authority /INVOLVE, which state:

*“You do not need to apply for ethical approval to involve the public in the planning or the design stage of research, for example helping to develop a protocol, questionnaire or information sheet, being a member of a research advisory group, or preparing an application for funding or ethical review, even when those people are approached for this role via the NHS … However, there are some situations where the involvement of the public may raise ethical concerns, for example, when they will be involved with collecting and analysing data, such as helping to analyse survey data, conducting interviews, facilitating focus groups or recruiting participants.” (Health Research Authority / INVOLVE, 2016, p2-3).*

It is problematic if youth and adult involvement activity is not going ahead in mental health/self-harm/suicide research due to unwarranted ethical concerns, particularly as this is something that ECRs may not feel in a position to challenge or debate. It is important to emphasise that there is increasing research evidence that participating in research on self-harm and suicide does not have a detrimental effect on participant well-being. A recent meta-analysis of the impact of exposure to suicide-related content in research protocols indicates a reduction in suicidal ideation and behaviour after being exposed to suicide-related content or suicide assessment. Notably, this reduction was largest in young people [21] There is no reason to assume involvement in such research in an advisory capacity should be any different (though future research could usefully address this).

The second barrier identified relates to the practical costs (time and money) that undertaking involvement work can entail (balanced against the perceived benefits) and the third relates to the challenges involved in recruiting people to be involved in research. ECRs have a limited timeframe in which to complete their research and face many other competing research priorities. However, successful approaches to engaging young people in ‘sensitive’ involvement activities, for example in drug and alcohol research, requires a researcher to be flexible and young-person centred, and crucially takes time [17]. Nonetheless, within this context, the respondents highlighted potential ways in which to facilitate their youth and adult involvement work: expert examples, expertise and guidelines; and investment in involvement work (practical resources, especially money; wider community support; a supportive culture within institutions). In particular, funding needs were highlighted by ECRs – indeed, over two thirds of ECRs did not have access to funding to support their involvement activities. INVOLVE offer guidance regarding payment for PPI group members to compensate them for their time and expertise. It would be difficult for ECRs to adhere to these best practice guidelines without access to sufficient funds (e.g., where funding is not available, involvement activities may be reliant on people giving their time for free. Additionally, ECRs may be unable to cover travel costs incurred by involvement group member). This potentially magnifies already complex power imbalances, if some people (i.e. researchers) are being paid for their time and expertise, whilst others (i.e. PPI members) are not. The costs of involvement have previously been cited as a barrier to encouraging user involvement in mental health services [22], but there is evidence that the provision of small research development bursaries for PPI can support involvement activities and help develop this aspect of research [23]. However, much of the other support required could actually be provided at minimal cost to institutions. There is ample published guidance on PPI, but perhaps more hands-on mentoring or discipline specific guidance is required, or even simply improved signposting to such resources. Some of this could be achieved by raising the profile of research involvement as a valuable research activity within higher education research – although this would require significant buy-in from institutions.

At a more individual level we would urge ECRs to think creatively about how to best achieve their research involvement aims, by utilising technology and social media (i.e. virtual involvement) and by networking and collaborating with research colleagues in their field(s). In order to act as drivers for change, ideally ECRs need the support of their supervisors, senior colleagues and institutions. There is an apparent lack of clarity regarding ethical and risk governance issues surrounding involvement work in mental health, self-harm and suicide research, which may pose a substantial barrier to youth and adult involvement in this research field. We would argue there is a need for more targeted guidance for involvement work in this area and improved access to (modest) financial support.

### Limitations

The survey respondents constitute a highly selective and small sample, with an interest in youth and adult involvement work. Due to our recruitment strategy (social media) we do not know how many ECRs in mental health research chose not to participate. Future survey work could aim to recruit a larger sample who are not necessarily as invested in involvement work.

The terminology used to describe involvement work is confusing and used interchangeably (e.g. engagement, involvement, knowledge exchange, PPI). Although we attempted to be clear in our participant recruitment material and wording of questions, potential participants may not have considered themselves eligible to participate due to another term being used in their experience, or may not perceive the work they do to be involvement work (e.g. consulting an advisory committee). Again, the distinction between youth and adult involvement can also be variable and thereby impact how the two populations are considered.

## Conclusion

The involvement of youth and adult populations in mental health, self-harm and suicide research is reported to be valuable and important by researchers at an early stage in their career. Through supportive institutions and working relationships with senior researchers, our data suggest that ECRs are building their confidence and ability to conduct involvement within their own research and long-term projects. However, improved signposting and resources (such as funding opportunities and recruitment strategies) are necessary to develop the research culture so that involvement is not considered simply as tokenistic.

In addition, we feel it is important to acknowledge the barriers which challenge those engaging with youth involvement, particularly around ethical concerns and perceived risks. Due to the pressing clinical relevance of advancing our understanding of mental health, self-harm and suicide amongst young people, it is important that their voices are heard within research. This can then lead to the development of future research which is applicable, relevant and accessible to these populations.

## Abbreviations

ECRs: Early Career Researchers
NHS: National Health Service
NIHR: National Institute for Health Research
NRES: National Research Ethics Service
PPI: Patient and Public Involvement
UK: United Kingdom
